# Rhythmic coordination in rapid duets of little crakes: cooperative signalling without shared experience

**DOI:** 10.1098/rspb.2025.1100

**Published:** 2025-07-23

**Authors:** Jan Jedlikowski, Paweł Ręk

**Affiliations:** ^1^ Biological and Chemical Research Centre, Faculty of Biology, University of Warsaw, Warsaw, Poland; ^2^ Department of Behavioural Ecology, Institute of Environmental Biology, Faculty of Biology, Adam Mickiewicz University, Poznan, Poland

**Keywords:** vocal coordination, note-by-note coordination, entrainment, isochrony, duet, rhythm

## Abstract

Coordinated vocal displays are common in animals with long-term social bonds, reinforcing alliances and aiding territorial defence. However, they also occur in species with brief social ties and limited learning, raising questions about their strategic function. Little crakes (*Zapornia parva*) provide a useful model to explore this, as territorial pairs produce antiphonal duets of rapid trills despite short-term pair bonds. We investigated the structure, formation and function of little crake duets. Pairs produced rapid trills with strict note alternation and high isochrony, eliminating note overlap after a brief synchronization phase to maintain a stable rhythm. Playback of solo trills showed that, despite the high tempo, unpaired males spontaneously synchronized their notes with the playback, demonstrating that note alternation does not require prior interaction with a specific partner. A second experiment showed that temporally precise duets elicited stronger responses than irregular, overlapping ones, indicating that rhythmic regularity enhances signal efficacy. We conclude that coordination in little crake duets results from spontaneous rhythmic alignment rather than learned note-by-note adjustments. We suggest that precise alternation of notes, strictly enforced by the close proximity of the male and female, may, independently of shared experience, serve as an index of pairing, indirectly supporting the cooperative territorial defence.

## 1. Introduction

Coordinated vocal displays occur in species that form cooperative bonds of varying strength and duration [1–5]. In stable social groups, including long-term pairs, these displays often signal partnership strength to outsiders [6,7]. By demonstrating a high level of coordination and consistency, pairs or groups convey their unity, deterring rivals and reducing the risk of territorial challenges [6,8,9]. Yet, species engaged in short-lived cooperative interactions also exhibit vocal coordination [10–12], raising a question: if these alliances are brief, what strategic value does a signal that emerges so quickly actually have? Understanding this could shed light on the broader role of coordination in social communication.

Vocal coordination arises from different proximate mechanisms shaped by social dynamics as well as physical constraints on signal transmission [4,13,14]. In species with long-term pairings, coordination and consistency improve over time, relying on learning and real-time vocal modifications [7,8]. This process, known as note-by-note adjustment, involves fine-scale corrections to timing and structure [15–17], allowing for increasingly precise synchronization through repeated practice [6,8]. Initially imprecise, duet performance becomes more coordinated as vocal partners continuously learn and adapt to each other’s signals [8]. In contrast, species with brief social interactions often achieve synchronization through rhythmic entrainment, where vocal rhythms spontaneously align via interactions among internal oscillators [18]. This mechanism enables immediate and precise synchronization without requiring prior learning, often resulting in faster tempos and more consistent timing compared with note-by-note coordination [19–21]. However, this comes at a cost: entrainment requires a highly regular reference rhythm to which individuals can align [19]. Humans, for example, rely on rhythmic entrainment in activities such as clapping in unison, marching or performing coordinated music and dance, all of which depend on a steady, predictable beat [22]. High-tempo synchronization is also subject to physical constraints: the faster the rhythm, the more disruptive even slight transmission delays become [13]. Just as musicians in an orchestra struggle with timing when spread far apart, animals producing rapid, precisely timed vocalizations must remain close together to ensure their signals reach the receiver in the intended temporal structure [13,19]. This limitation suggests that when an observer hears a perfectly coordinated, high-speed duet, it is strong evidence that the performers are physically near each other.

The function of vocal coordination ultimately depends on whether it reinforces long-term social bonds or facilitates short-term cooperative interactions. In species with enduring bonds, such as duetting songbirds, repeated practice enhances coordination, making duet performance a reliable social signal. This is seen in Australian magpie-larks (*Grallina cyanoleuca*), where improved coordination signals pair cohesion and helps deter territorial intrusions [6]. In contrast, highly coordinated collective displays of insects and amphibians, like the choruses of neotropical katydids (*Neoconocephalus spiza*) or Japanese treefrogs (*Hyla japonica*), rely on immediate and precise synchronization without requiring learning or prior interaction [23,24]. However, it is unclear whether such displays are truly synergistic and adaptive at the group level. They may emerge as a by-product of individual behaviours rather than a shared communicative goal [19,25–27].

While clear evidence for spontaneous rhythmic entrainment remains lacking in higher vertebrates [19,25,28], some non-passerine birds challenge this pattern. Rails (Rallidae) and grebes (Podicipedidae) produce precisely synchronized trills in duets despite lacking vocal learning and forming only brief pair bonds [29–31]. The mechanisms underlying this coordination remain unclear, yet their duet tempo can reach nearly 30 Hz (see §3), making them among the fastest known coordinated signals in nature [19]. These displays emerge during territorial interactions, suggesting a cooperative function, even in the absence of long-term pair bonds and vocal learning [32,33]. Notably, their structure and spontaneous emergence more closely resemble the unlearned choruses of amphibians and insects rather than the learned coordination of songbirds.

**Figure 1.**
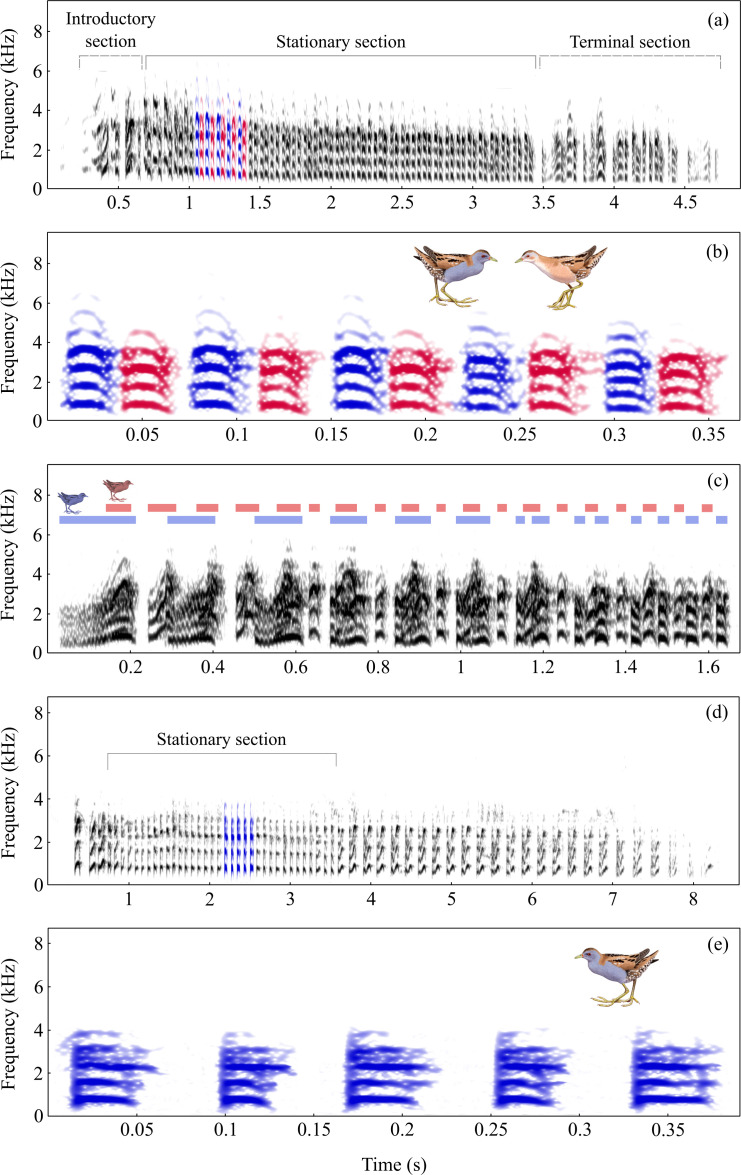
Spectrogram of the little crake duet (a–c) and solo trills (d,e). Panels (a) and (d) show the entire duet and solo, panels (b) and (e) show magnified fragments of stationary sections of the duet and solo, and panel (c) shows the introductory section of the duet. Colours indicate notes of different individuals. Illustration of a pair of little crakes by Szabolcs Kókay.

We studied the coordination of trill duets in little crakes (*Zapornia parva*), a highly territorial species in which pair bonds typically last for a single breeding season [32]. Both males and females produce rapid trills, either solo or in duets composed of dozens of regularly alternating notes (figure 1). Despite their high temporal precision, little crakes achieve this coordination almost immediately after pairing. Perfectly timed duets can be heard before egg-laying begins (J. Jedlikowski, 2024, personal observation), suggesting that their ability to coordinate does not require prior experience or training with a specific partner. This immediate synchronization may serve as a cooperative signal, reinforcing the perception of a unified pair. Hearing a regularly alternating duet at such a high tempo, without overlapping notes, might indicate that the territory is defended by a cooperating pair rather than two independent individuals. Because maintaining perceived regularity requires both sound sources to be in close proximity [13], this physical constraint may reinforce the duet’s role as a social signal, providing a reliable indicator of proximity and cooperation, even in species with ephemeral pair bonds.

To examine the function of vocal coordination in little crake duets, we analysed the structure of natural duets and solo trills, elicited responses to artificial solo trills and conducted a territorial playback experiment. First, we assessed the temporal organization of natural trills, focusing on their regularity and the degree of overlap between male and female vocalizations, as both isochrony and clear alternation are essential for rhythmic coordination. Next, we tested whether individuals could spontaneously synchronize with an unfamiliar and extremely fast solo trill, forming a precisely timed duet without overlapping notes, which would indicate a capacity for coordination that does not depend on learning or prior partner experience. Finally, we examined whether regular alternation influences territorial interactions by testing if clearly structured regular duets provoke stronger responses from territorial pairs than irregular, overlapping ones. If precise coordination signals alliance and joint territorial defence, crakes should respond more strongly to well-timed duets.

## 2. Material and methods

### (a) Study area and species

The study was conducted in the Mazurian Lakeland (northeast Poland), where little crakes breed from April to September. They inhabit dense emergent vegetation across various aquatic ecosystems, from large lakes to small midfield waterbodies [34]. Data were collected from 20 such sites (mean area: 2.99 ha ± 0.58 s.e., range: 0.5−9.1 ha), located between 53°48ʹ−54°17ʹ N and 21°27ʹ−21°42ʹ E. Spontaneous trill recordings were gathered in May–June 2016−2017 and 2024, the playback experiment was conducted in May–June 2022−2024, and artificial solo trill stimulation took place in May–June 2024.

Little crakes use various vocal signals to communicate with partners, neighbours and intruders. During territorial interactions, they produce a limited repertoire of simple, innate calls, including trills [35,36]. This study focuses exclusively on trills, as they can be performed solo or as duets by mated pairs (figure 1; see electronic supplementary material, videos S1 and S2).

### (b) Natural observations

We analysed the temporal structure of natural solos and duets to assess their regularity and coordination, expecting little crakes to match their isochronous rhythms to avoid note overlap. For rhythms to be coordinated and overlap-free, they must form a predictable reference framework for a potential partner; that is, the trill must be evenly spaced and slow enough to provide gaps into which the partner’s notes can fit. Therefore, trills in solos should exhibit a similar degree of isochrony and tempo as those in duets. High isochrony is thus a necessary condition for rhythmic entrainment, but it is insufficient on its own. Some form of active coordination appears necessary in duets, even when rhythmic entrainment is involved, because even small stochastic timing errors can accumulate over time, leading to note overlap if the sequence is long enough [37]. These errors are typically addressed through note-by-note correction, where each individual note is adjusted for timing inconsistencies. In contrast, similar overlap can arise from even minor discrepancies in tempo between individuals [37]. However, limiting overlap due to tempo discrepancies requires adjustments at the macro level, a crucial aspect of rhythmic entrainment. In little crakes, the latter mechanism should therefore have a much greater potential to cause overlap.

To examine these predictions, we recorded spontaneous trills using a Sennheiser ME 67 directional microphone and a Marantz PMD 661 MKII digital recorder (PCM: 48 kHz, 16 bit) at distances of less than 5 m. We focused on the central stationary sections of duet trills and stationary sections of solo trills, which consist of parts with a stable tempo and monotonous notes (see figure 1). From the recordings, we extracted 26 stationary sections from duets produced by 20 pairs and seven stationary sections from solos produced by five birds. Although the sex of the callers was unknown, we could visually assign the notes to a specific caller throughout the spectrogram, enabling us to measure the trills of individual birds.

All temporal measurements were taken from the waveform, with spectrograms used only for better orientation within the recording. The recordings were analysed in Raven Pro 1.6 software. The isochrony was defined based on two parameters: the coefficient of variation (CV) and the normalized pairwise variability index (nPVI). CV was calculated using the formula


$$CV=\frac{s.d.}{\bar{x}}\times 100\left(\%\right),$$

where s.d. and $\bar{x}$ refer to the lengths of the intervals measuring the time between the beginnings of successive notes of an individual (first-order differenced intervals). nPVI was calculated using the following equation:


$$nPVI=\;\frac{100}{m-1}\times \sum_{k=1}^{m-1}\left|\frac{d_{k}-d_{k+1}}{\frac{1}{2}\left(d_{k}+d_{k+1\;}\right)}\right|,$$

where *d*_*k*_ indicates the length of the *k*th first-order differenced interval and *m* indicates the total number of these intervals. These two measures are complementary, capturing distinct aspects of temporal variability. CV reflects total variability, encompassing both short-term fluctuations and slower changes over longer time scales, while nPVI focuses on variability between successive intervals and is more sensitive to local timing deviations, with reduced sensitivity to long-term trends. Therefore, CV is frequently used in studies of group signalling in insects, where isochrony is typically very high [19], whereas nPVI is more frequently employed in research on rhythmic patterning in speech and music, as well as in studies of communication of higher vertebrates [38,39]. The nPVI is a dimensionless measure, and values below 20 are generally interpreted as indicating high isochrony [39]. For duets, isochrony was measured separately for each individual across the entire central stationary section. In the case of solos, only the maximum isochrony was measured, based on the most regular segment of 10 consecutive intervals, as stationary sections in solos are much shorter than those in duets (see §3).

### (c) Simulation-based approach to overlap analysis

We simulated the variability of first-order interval lengths and tempo in duets to assess which of these parameters contributes more to the coordination of trills in duets. To this end, we conducted two independent permutation tests, each with 500 iterations. In the test assessing the impact of interval variability during trills, we randomly swapped each interval with one selected from a range of ±3 intervals within the trill. This procedure preserved the overall tempo and long-term trends of the trill but modified the relationships between adjacent intervals. In the test evaluating the impact of trill tempo, we randomly adjusted the tempo of the trill by either lengthening or shortening all the intervals within a trill by a constant value sampled from a normal distribution. The mean and standard deviation of this distribution were based on all analysed natural trills from duets. This manipulation preserved the variability of the intervals within the trill but altered its overall length while keeping it within a natural range. For each duet, only one randomly selected trill underwent permutations, while the second trill served as a reference to assess overlap with the permuted trill in each of the 500 iterations. These manipulations altered the temporal relationships between the trills of duet partners. In both tests, the null hypothesis stated that the proportion of iterations with an overlap level equal to or lower than that observed in natural duets should be greater than 5%. Therefore, a significant result would indicate that achieving the same precision as in natural duets is unlikely following the given manipulation. These tests were conducted in Excel 2016 (the VBA code used for simulations is provided as the electronic supplementary material).

### (d) Stimulation with artificial solo trills

We investigated whether solitary little crakes could spontaneously join an artificial solo trill, forming a precisely timed duet without prior experience with the stimulus. If duet coordination does not require prior experience with a specific partner, birds should alternate their notes without overlap while maintaining the rhythm of the artificial trill.

To create the playback stimulus, we selected a high-quality natural duet in which the partners alternated their notes without overlap in the stationary section. The recording was high-pass filtered (0.3 kHz), and the notes of one bird were systematically muted throughout the entire sequence. In the introductory and terminal sections, only non-overlapped notes were removed. This modified recording was then used as the playback stimulus, prepared using Avisoft SASLab Pro (v. 5.3.2−36). The trill contained nearly 150 notes in the stationary section, making error-free synchronization highly improbable by chance alone.

We played this artificial solo to unpaired little crakes, placing a loudspeaker (JBL Charge Essential 2, 60–20 kHz) on a floating platform hidden in dense vegetation near a calling solitary bird. Since birds only joined the solo trill when close to the speaker, we initially played female-specific mating calls to attract unmated males and switched to the trill once the bird approached. Each responder was visually confirmed before playback began. The stimulus was played from a smartphone running a WAV player for Android, connected via Bluetooth to the speaker. The recordings were made 5 m from the loudspeaker using the same equipment and analysis methods as for natural duets. We measured the isochrony (CV and nPVI) of the joined trill and quantified the percentage of playback notes overlapped by the bird’s notes, considering only the section where an actual duet was formed.

### (e) Playback experiment—design

We conducted an acoustic playback experiment to test whether the regularity of duet trills influences territorial responses. Because the fast tempo of little crake duets makes them susceptible to note overlap when signallers are spatially separated [13], a precisely timed duet may serve as a reliable signal of alliance and joint territorial defence. If such coordination reinforces the perception of a cooperating pair, regular duets should provoke stronger reactions from territorial pairs than irregular, overlapped ones.

The experiment targeted 20 mated pairs, each of which was subjected to two playback treatments. In the regular treatment, birds heard a duet trill in which the partners’ notes alternated without overlap, while in the irregular treatment, they heard a duet with partially overlapping notes. Such overlaps occur naturally but are rare. Each pair received both playbacks, derived from the same original duet recording to control for individual variation. In total, we prepared 40 playbacks using 20 unique duet trills from 17 pairs, recorded in our study population 5−7 years earlier with the same recording technique described previously.

To prepare the stimuli, we followed the same approach as for artificial solo trills. We selected duets in which partners’ notes alternated without overlap in the stationary section, and high-pass filtered the recordings (0.3 kHz). Then, we created two copies of each duet and systematically removed the notes of one individual in each copy. By later merging both tracks into a stereo file, we obtained a recording in which the male and female trills were assigned to separate channels, serving as the stimulus for the regular treatment. For the irregular treatment, we shifted the notes of one bird by −30 ms relative to the other, mimicking the effect of a spatial separation of approximately 9.5 m (assuming a sound speed of 314 m s^−1^). As a result, the overlapping portions of the trill averaged 17.5 ms.

### (f) Playback experiment—field methods

Before the experiment, we monitored each waterbody at least once a week, from the arrival of little crakes, mapping territories and locating nests. Only pairs of incubating eggs were tested, ensuring stable territorial conditions. To prevent retesting the same individuals, all birds from tested pairs were captured and banded after the experiment in the first two years, while in the third year, we only checked for the absence of bands.

Each pair received both playback treatments in a balanced order (10 pairs heard the regular playback first; 10 heard the irregular playback first), with a 60 min interval between treatments. Playback sessions were conducted between 05.30−09.00 and 19.00−21.00 (local time). Each session lasted 7.5 min, following a structured playback pattern: two stimuli followed by 1 min of silence, one stimulus followed by 1 min of silence and a final stimulus followed by 5 min of silence. The maximum amplitude was calibrated to 80 ± 1 dB at 1 m using a sound level meter (UT-352 UNI-T), corresponding to natural duet amplitudes measured in the field.

For playback, we used the same stereo loudspeaker and player as in the artificial solo trill playback, ensuring that the two channels were played through separate speaker membranes. The loudspeaker was placed on a floating platform, hidden in dense vegetation approximately 10 m from the nest in a random direction, 1 h before the first playback. This distance reflected the typical territory size defended by little crakes [40].

### (g) Responses to experimental treatments and statistical analysis

We measured both vocal and non-vocal responses of breeding pairs to playback. During territorial interactions, little crakes typically approach rivals and increase calling activity [35,36]. We assessed territorial response using three measures: latency to the first call as a measure of readiness to defend the territory, the number of calls produced and a binary yes/no on approach to within 1.5 m of the speaker as complementary measures of aggressiveness.

Vocal responses were analysed at the pair level, while non-vocal responses were assigned to individuals. We recorded vocal responses with four omnidirectional microphones (Sennheiser ME62) placed in the territory and a Roland R-44 solid-state field recorder (48 kHz, 16-bit). Non-vocal behaviours were captured with two Bushnell NatureView Cam HD camera traps positioned near the loudspeaker. While morphological differences allowed the identification of individuals approaching the speaker, the similar vocal repertoires of males and females prevented the assignment of calls to specific individuals.

We analysed the responses of mated pairs to regular and irregular duets using generalized estimating equations (GEE). This method is suitable for non-normally distributed variables with repeated measurements [41]. Three independent analyses were conducted for the three response variables. The likelihood of the approach was analysed using a binomial distribution with a logit link function. Latency to the first call and number of calls, both zero-truncated variables, were analysed using a negative binomial distribution with a logarithm link function. Post hoc comparisons were performed using Fisher’s LSD method to generate confidence intervals and compare treatment means. Statistical analyses were conducted in SPSS 29. All *p*-values were two-tailed, and results are reported as means ± s.e.

## 3. Results

### (a) Natural duet trills

Little crake duets followed a consistent structural pattern across all recorded pairs, with strict alternation between partners throughout the central section (figure 1a,b). The short introductory section (0.6 ± 0.05 s) exhibited characteristics of gradual coordination between partners. The notes were initially long and overlapped but gradually shortened until they became fully separated (figure 1c). This marked the beginning of the central and longest section (3.1 ± 0.22 s), where the partners’ notes were short (23 ± 0.1 ms) and strictly alternated without overlap (figure 1b). This section often exceeded 100 notes (86 ± 5.7; range 24−144) and reached an extremely fast tempo (27.4 ± 0.22 Hz; range 24.8−29.2 Hz for two birds in a duet), meaning that the intervals between a note of one individual and the following note of its partner were only 13.8 ± 0.50 ms. The trills in this section were produced with high isochrony, as indicated by low CV and nPVI of first-order differenced intervals (CV = 4.68 ± 0.18%, range: 2.34−8.05%; nPVI = 4.83 ± 0.24, range: 2.41−10.10; *n* = 52 trills from 26 duets), and complete absence of note overlap. The absence of overlap was reflected in the almost identical tempos of males and females within a duet compared with randomly paired trills from different duets (mean tempo difference within duet 0.04 ± 0.01 Hz, between-duet variation s.d. = 0.8 Hz; *t*‐test: *t*_50_ = 9.54, *p* < 0.001), as well as in nearly identical first-order differenced interval lengths (mean interval difference within duet 0.2 ± 0.05 ms, between-duet variation s.d. = 3.19 ms; *t*‐test: *t*_50_ = 10.27, *p* < 0.001). In the permutation tests, this level of precise coordination was achieved in 12.6% of iterations with randomized intervals and in just 0.8% of iterations with randomized tempo. High coordination is therefore much less likely when duet partners differ in tempo than when they make stochastic timing errors (Fisher’s exact test: *p* < 0.001). The transition into the terminal section (1.1 ± 0.27 s) was characterized by lengthening silent intervals, a decline in tempo and the reappearance of note overlap, leading to a loss of temporal regularity (figure 1a).

The solos did not follow a schematic structure like the duets but instead consisted of short stationary sections of fast trills (2.2 ± 0.36 s), separated by segments with irregular intervals and note structures (figure 1d,e). However, even within these stationary sections, trills were produced with high isochrony (local isochrony: CV = 2.96 ± 0.42%, range: 1.33−5.08%; nPVI = 3.29 ± 0.74, range: 1.51−7.43; *n* = 7 trills) and at tempos comparable to those of individual birds in duets (14.95 ± 0.27 Hz; range: 14.14−15.91 Hz).

### (b) Artificial solo trills

**Figure 2.**
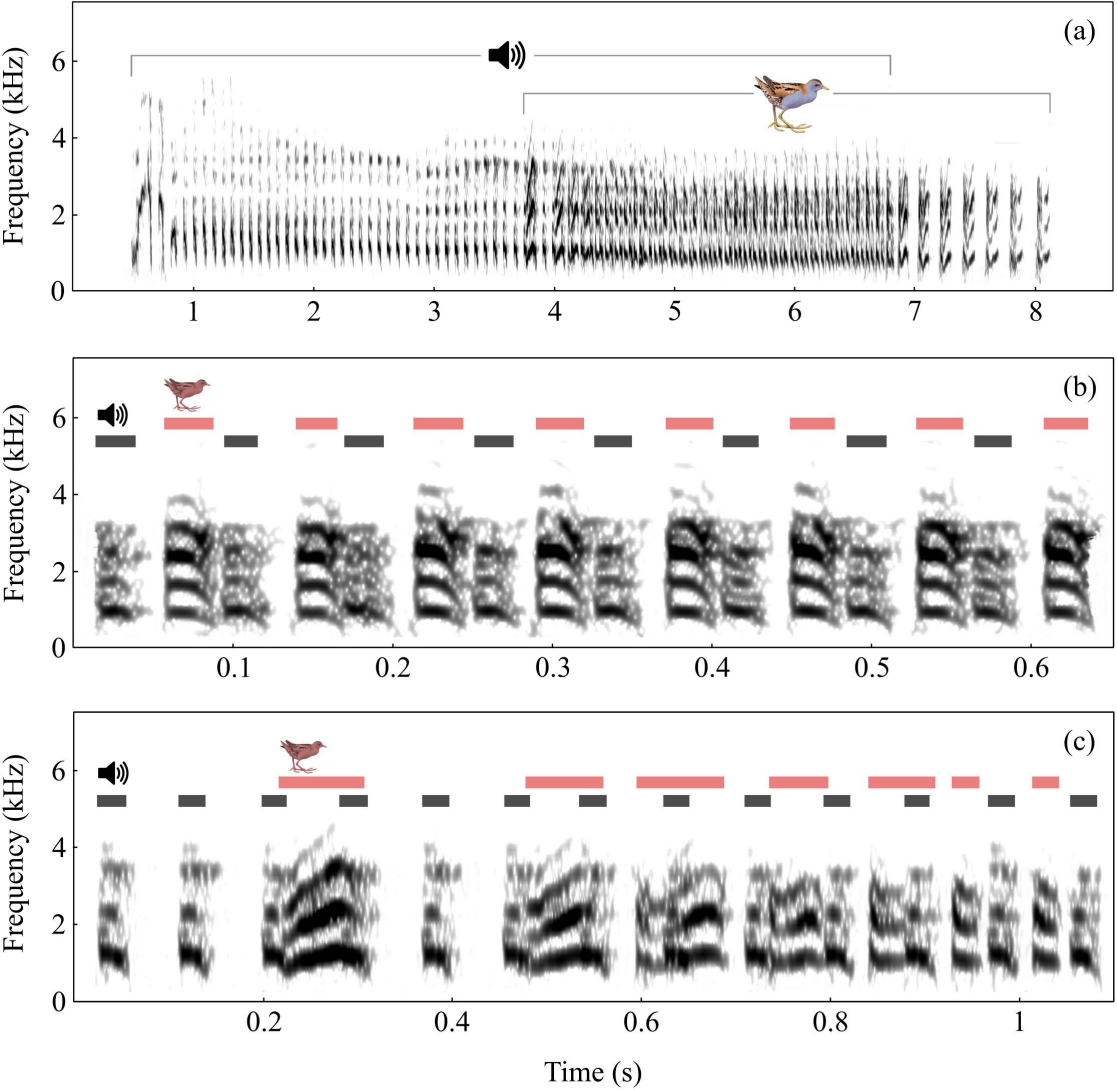
(a) Spectrogram showiing the vocal interaction of the male with the trill of the artificial solo playback. (b,c) Magnification of the stationary section (b) and the introductory section (c). Red lines indicate notes uttered by birds, and black lines indicate playback notes.

The birds precisely joined and followed the calling rhythm of the natural playback (figure 2a,b). A total of nine birds were exposed to artificial solo trills, and five of them joined 15 playbacks (1–6 per bird). These five birds always joined after the playback started (2.4 ± 0.39 s). Their responses included a short initial phase (0.2 ± 0.06 s), during which notes were longer and slightly overlapped before becoming fully separated (figure 2c). When joining the playback, their trills were characterized by high isochrony (CV = 5.87 ± 0.33%, range: 3.75–8.59%; nPVI = 7.48, range = 4.00–12.77) and minimal overlap of notes (0.25% ± 0.12% of total note length), closely matching the pattern of the playback’s calls. The state of near-perfect alternation lasted for 2.7 ± 0.35 s, corresponding to 11–63 notes of the playback.

### (c) Playback experiment

**Figure 3.**
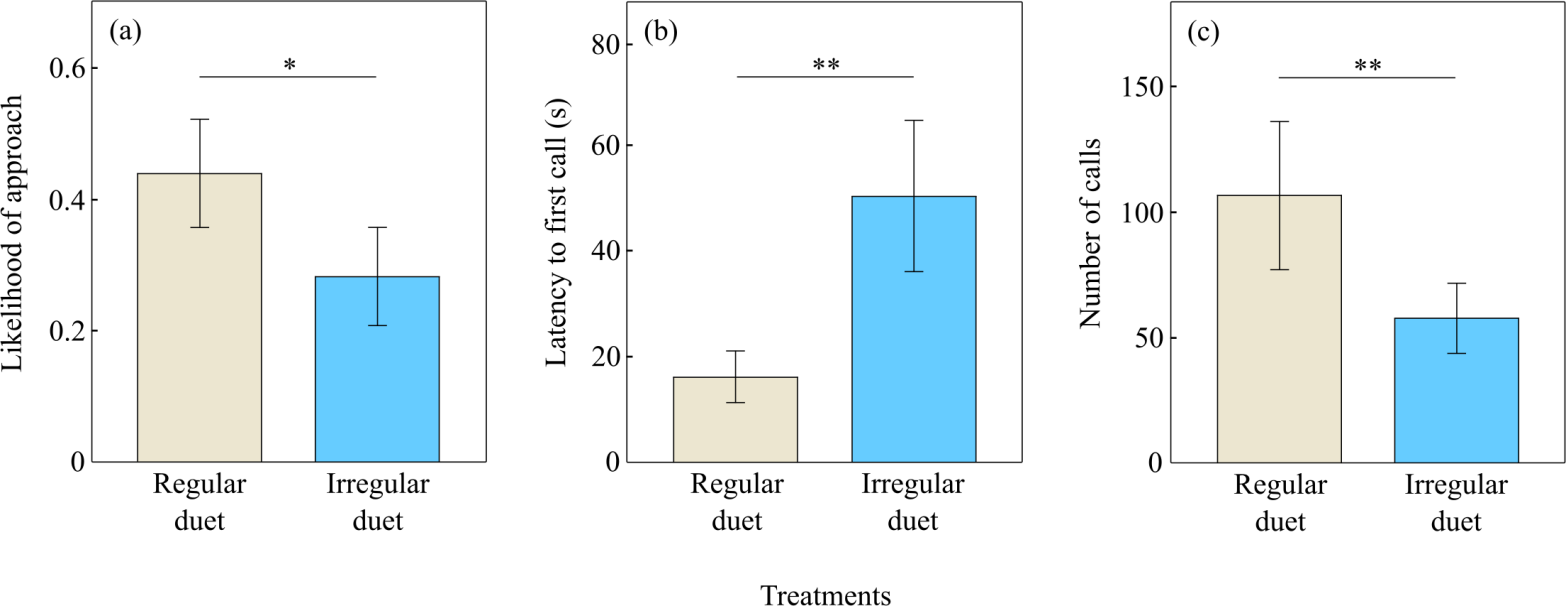
(a) Non-vocal and (b,c) vocal responses of little crakes to treatments. The bars show mean ± s.e. Significant differences between responses to treatments are indicated by *p < 0.05; **p < 0.01.

The high regularity of alternation and lack of overlaps were important for duet efficacy. When regular trill duets were played, little crakes were more likely to approach the speaker (GEE: Wald *Χ*^2^_1_ = 5.03, *p* = 0.025; figure 3a), responded vocally faster to the playback (GEE: Wald *Χ*^2^_1_ = 6.96, *p* = 0.008; figure 3b) and produced more vocalizations overall (GEE: Wald *Χ*^2^_1_ = 6.20, *p* = 0.013; figure 3c) compared with when irregular trill duets were played.

## 4. Discussion

Our study shows that little crake pairs synchronize spontaneously, maintaining a consistent rhythmic pattern that plays a key role in social signalling. Duets formed when pairs produced regular, isochronous trills and strictly alternated their notes without overlap, creating a stable temporal structure. Additionally, solitary birds coordinated their trills with the isochronous trills of unfamiliar individuals, demonstrating that such coordination emerged spontaneously without shared experience. Finally, territorial pairs responded more strongly to well-timed duets than to irregular, overlapping ones, highlighting the importance of precise timing in communication between pairs. Our results suggest that, due to the constraints of fast, non-overlapping note alternation, well-coordinated duets reliably indicate that partners are in close proximity and may also reflect joint engagement in territorial defence.

Pairs initiated duets by progressively aligning their isochronous trills, gradually eliminating overlap until they established a stable note alternation. This initial alignment suggests that exposure to a partner’s rhythmic signals facilitates coordination. Such a process shares characteristics with auditory priming [42], where prior exposure to rhythmic patterns enhances neural synchronization and improves temporal coordination between individuals. Permutation tests further support this by showing that, to maintain low levels of overlap, individuals must primarily coordinate their tempos. Once a shared tempo is established, coordination remains stable even if small stochastic errors occur between notes, as these have minimal impact on overall timing. As the duet progresses into the central section, rhythm stabilizes and no further major alignment is necessary, although both males and females should continue adjusting their tempos to maintain synchronization. High isochrony and strict note alternation during this phase indicate that the pair locks into a coordinated temporal pattern. In the terminal phase, both partners gradually slow their tempos, leading to a desynchronization of their trills and a breakdown in strict alternation.

Instead of relying on moment-to-moment adjustments, the structure of little crake duets aligns with characteristics predicted for entrainment mechanisms [19]. Studies on reaction times in animals [43,44] suggest that the tempo in the central section of the duet may exceed the limits of real-time interactive response, making note-by-note coordination unlikely. Therefore, trilled vocalizations in birds, including little crakes, typically involve rapid, rhythmically stable syllable repetitions driven by automatic motor patterns rather than flexible control of individual notes [45]. These findings are consistent with our permutation test results, which suggest that during the central phase, both partners rely on tempo adjustments to avoid overlapping, eliminating the need for precise per-note corrections. Additionally, the tempo and isochrony of solo trills in little crakes, which closely match those in duets, suggest that individuals are capable of producing stable rhythmic patterns independently. This reinforces the idea that duet coordination arises from rhythmic alignment rather than continuous mutual adjustments.

The fact that unpaired individuals spontaneously alternated their notes with unfamiliar birds without prior interaction suggests that duet coordination in little crakes does not rely on training with a specific partner. Unlike species in which duet precision develops through common experience [6,7], little crakes did not require prior practice to achieve coordination. In our trials, solitary birds adjusted almost immediately, sustaining flawless note alternation for dozens of repetitions, making it highly unlikely that such coordination emerged by chance alone. This suggests that the ability to coordinate may be intrinsic rather than learned. Moreover, the successful alternation between unfamiliar individuals indicates that duet coordination does not depend on prior pair bonding or social familiarity, as seen in some primates and songbirds [6,46–48]. Similar spontaneous coordination has been observed in other taxa, such as fireflies flashing in unison or vibrational duetting in green lacewings (*Chrysoperla plorabunda*) [43,49], where rhythmic behaviours emerge without prior interaction. The consistency of note alternation over many cycles further implies that the underlying mechanism is governed by a stable rhythmic structure, preventing gradual drift, as random variation would likely lead to misalignment over time [22]. These findings provide additional support for our earlier conclusions on natural duets, reinforcing the idea that little crake note alternation results from a process of rhythmic alignment rather than continuous real-time adjustments.

The regular alternation of male and female trills in a duet enhanced its communicative function. Our results showed that little crakes not only distinguished between duets with regular and irregular timing but also responded more strongly to well-coordinated alternation, indicating that precise timing carried important social information. Similar sensitivity to temporal structure has been demonstrated in other bird species [2,14,50]. The absence of overlapping notes in natural duets is therefore unlikely to be incidental but rather serves an adaptive function. One key constraint shaping this function is the high tempo of the duet, which requires partners to remain in close proximity to maintain precise alternation [13]. Our audio-video recordings confirmed that duetting little crakes were indeed positioned next to each other, reinforcing the idea that duet structure signals that the performers are a mated pair rather than two unrelated individuals. Furthermore, because little crakes are highly territorial, such well-timed duets are unlikely to occur between competitors, signalling both pairing and cooperation. This physical constraint makes duet coordination a strong example of an index signal of partners’ proximity [51], one that is inherently honest because it cannot be produced by unpaired individuals or those lacking the motivation to form a pair. The ability of solitary males to integrate into an ongoing duet, observed when they responded to solo playback, further supports this interpretation. By first attracting them with female calls, we created a context in which their subsequent response to solo playback reflected an active intent to ally rather than an antagonistic territorial reaction. These findings support the idea that the structure of little crake duets reliably encodes social information, with precise note alternation serving as a key signal of both pair status and cooperative intent.

While many animals produce coordinated vocalizations [52–56], little crakes stand out by spontaneously achieving precise rhythmic alternation to produce a structured social signal. In many species, coordinated signals emerge through gradual refinement, requiring social experience or active adjustments between individuals [6,7,57]. In others, precise timing is present, but it remains unclear whether coordination reflects cooperation or merely individual adaptation to shared rhythmic patterns [26]. In contrast, little crake duets reach near-perfect synchronization from the outset, suggesting a mechanism that enables immediate coordination. Moreover, the high tempo and strict non-overlapping structure of their duets impose natural constraints that make them an inherently honest signal of pair proximity and cooperative engagement, particularly in territorial defence. This ability to spontaneously generate a well-structured and socially meaningful vocal signal without prior interaction is a feature never documented in birds. These characteristics set little crake duets apart from most known vocal coordination systems, illustrating how spontaneous rhythmic alignment can serve as a robust mechanism for social signalling in a territorial context.

## Data Availability

The dataset supporting this study has been deposited in the AMUReD research data repository [58]. Supplementary material is available online [59].

## References

[1] Mann N, Dingess K, Barker K, Graves J, Slater P. 2009 A comparative study of song form and duetting in neotropical Thryothorus wrens. Behaviour **146**, 1–43. (10.1163/156853908X390913)

[2] Diniz P, Ramos DM, Webster MS, Macedo RH. 2021 Rufous horneros perceive and alter temporal coordination of duets during territorial interactions. Anim. Behav. **174**, 175–185. (10.1016/j.anbehav.2021.02.007)

[3] Hall ML. 2009 A review of vocal duetting in birds. Adv. Stud. Behav. **40**, 67–121. (10.1016/S0065-3454(09)40003-2)

[4] Farabaugh SM. 1982 The ecological and social significance of duetting. In Acoustic communication in birds (eds DE Kroodsma, EH Miller), pp. 85–124. New York, NY: Academic Press.

[5] Vanderhoff EN, Bernal Hoverud N. 2022 Perspectives on antiphonal calling, duetting and counter-singing in non-primate mammals: an overview with notes on the coordinated vocalizations of bamboo rats (Dactylomys spp., Rodentia: Echimyidae). Front. Ecol. Evol. **10**. (10.3389/fevo.2022.906546)

[6] Hall ML, Magrath RD. 2007 Temporal coordination signals coalition quality. Curr. Biol. **17**, R406–R407. (10.1016/j.cub.2007.04.022) 17550763

[7] Keenan EL *et al*. 2020 Breeding season length predicts duet coordination and consistency in Neotropical wrens (Troglodytidae). Proc. R. Soc. B **287**, 20202482. (10.1098/rspb.2020.2482) PMC7779517 33323080

[8] Rivera-Cáceres KD, Quirós-Guerrero E, Araya-Salas M, Searcy WA. 2016 Neotropical wrens learn new duet rules as adults. Proc. R. Soc. B **283**, 20161819. (10.1098/rspb.2016.1819) PMC5136587 27881746

[9] Cuthbert JL, Mennill DJ. 2007 The duetting behavior of pacific coast plain wrens. Condor **109**, 686–692. (10.1650/8234.1)

[10] Legett HD, Page RA, Bernal XE. 2019 Synchronized mating signals in a communication network: the challenge of avoiding predators while attracting mates. Proc. R. Soc. B **286**, 20191067. (10.1098/rspb.2019.1067) PMC6790779 31594513

[11] Bailey WJ. 2003 Insect duets: underlying mechanisms and their evolution. Physiol. Entomol. **28**, 157–174. (10.1046/j.1365-3032.2003.00337.x)

[12] Greenfield MD. 1994 Synchronous and alternating choruses in insects and anurans: common mechanisms and diverse functions. Am. Zool. **34**, 605–615. (10.1093/icb/34.6.605)

[13] Ręk P, Magrath RD. 2020 Visual displays enhance vocal duet production and the perception of coordination despite spatial separation of partners. Anim. Behav. **168**, 231–241. (10.1016/j.anbehav.2020.08.002)

[14] Ręk P, Magrath RD. 2023 The quality of avian vocal duets can be assessed independently of the spatial separation of signallers. Sci. Rep. **13**, 16438. (10.1038/s41598-023-43508-w) 37777561 PMC10543378

[15] Fortune ES, Rodríguez C, Li D, Ball GF, Coleman MJ. 2011 Neural mechanisms for the coordination of duet singing in wrens. Science **334**, 666–670. (10.1126/science.1209867) 22053048

[16] Logue DM, Chalmers C, Gowland AH. 2008 The behavioural mechanisms underlying temporal coordination in black-bellied wren duets. Anim. Behav. **75**, 1803–1808. (10.1016/j.anbehav.2007.10.036)

[17] Hoffmann S *et al*. 2019 Duets recorded in the wild reveal that interindividually coordinated motor control enables cooperative behavior. Nat. Commun. **10**, 2577. (10.1038/s41467-019-10593-3) 31189912 PMC6561963

[18] Murphy MA, Thompson NL, Schul J. 2016 Keeping up with the neighbor: a novel mechanism of call synchrony in Neoconocephalus ensiger katydids. J. Comp. Physiol. A **202**, 225–234. (10.1007/s00359-016-1068-1) 26809565

[19] Greenfield MD, Merker B. 2023 Coordinated rhythms in animal species, including humans: entrainment from bushcricket chorusing to the philharmonic orchestra. Neurosci. Biobehav. Rev. **153**, 105382. (10.1016/j.neubiorev.2023.105382) 37673282

[20] Benichov JI, Globerson E, Tchernichovski O. 2016 Finding the beat: from socially coordinated vocalizations in songbirds to rhythmic entrainment in humans. Front. Hum. Neurosci. **10**, 255. (10.3389/fnhum.2016.00255) 27375455 PMC4893489

[21] Bouwer FL. 2022 Neural entrainment to auditory rhythms: automatic or top-down driven? J. Neurosci. **42**, 2146–2148. (10.1523/jneurosci.2305-21.2022) 35296536 PMC8936579

[22] Jacoby N, Polak R, London J. 2021 Extreme precision in rhythmic interaction is enabled by role-optimized sensorimotor coupling: analysis and modelling of West African drum ensemble music. Phil. Trans. R. Soc. B **376**, 20200331. (10.1098/rstb.2020.0331) 34420391 PMC8380984

[23] Greenfield MD, Roizen I. 1993 Katydid synchronous chorusing is an evolutionarily stable outcome of female choice. Nature **364**, 618–620. (10.1038/364618a0)

[24] Ota K, Aihara I, Aoyagi T. 2020 Interaction mechanisms quantified from dynamical features of frog choruses. R. Soc. Open Sci. **7**, 191693. (10.1098/rsos.191693) 32269798 PMC7137965

[25] Wilson M, Cook PF. 2016 Rhythmic entrainment: why humans want to, fireflies can’t help it, pet birds try, and sea lions have to be bribed. Psychon. Bull. Rev. **23**, 1647–1659. (10.3758/s13423-016-1013-x) 26920589

[26] Greenfield MD, Honing H, Kotz SA, Ravignani A. 2021 Synchrony and rhythm interaction: from the brain to behavioural ecology. Phil. Trans. R. Soc. B **376**, 20200324. (10.1098/rstb.2020.0324) 34420379 PMC8384058

[27] Greenfield MD, Schul J. 2008 Mechanisms and evolution of synchronous chorusing: emergent properties and adaptive functions in Neoconocephalus katydids (Orthoptera: Tettigoniidae). J. Comp. Physiol. **122**, 289–297. (10.1037/0735-7036.122.3.289) 18729657

[28] Roeske TC, Tchernichovski O, Poeppel D, Jacoby N. 2020 Categorical rhythms are shared between songbirds and humans. Curr. Biol. **30**, 3544–3555. (10.1016/j.cub.2020.06.072) 32707062 PMC7511425

[29] Konter A. 2014 Courtship and aggressive behavior of the least grebe in the breeding season. Wilson J. Ornithol. **126**, 140–147. (10.1676/13-161.1)

[30] Depino EA, Areta JI. 2020 Interspecific territoriality despite vocal divergence in two sympatric Laterallus crakes. J. Ornithol. **161**, 409–420. (10.1007/s10336-019-01735-x)

[31] Stiles FG, Levey DJ. 1988 The gray-breasted crake (Laterallus exilis) in Costa Rica: vocalizations, distribution, and interactions with white-throated crakes (L. albigularis). Condor **90**, 607–612. (10.2307/1368349)

[32] Taylor B. 1998 Rails: a guide to the rails, crakes, gallinules and coots of the world. New Haven, CT: Yale University Press.

[33] Ogilvie M, Rose C. 2003 Grebes of the world. Uxbridge, UK: Bruce Coleman.

[34] Jedlikowski J, Chibowski P, Karasek T, Brambilla M. 2016 Multi-scale habitat selection in highly territorial bird species: exploring the contribution of nest, territory and landscape levels to site choice in breeding rallids (Aves: Rallidae). Acta Oecol. **73**, 10–20. (10.1016/j.actao.2016.02.003)

[35] Jedlikowski J, Polak M, Brambilla M, Ręk P. 2021 Vocal and non-vocal behavior interact differently in territorial strategies of two sympatric Rallidae species. J. Ornithol. **162**, 243–254. (10.1007/s10336-020-01808-2)

[36] Jedlikowski J, Polak M, Koperski P, Ręk P. 2021 Response to heterospecific calls in non‐passerine species: can two Rallidae species recognise each other based on their vocalisations? Ethology **127**, 710–719. (10.1111/eth.13208)

[37] Repp BH, Keller PE. 2004 Adaptation to tempo changes in sensorimotor synchronization: effects of intention, attention, and awareness. Q. J. Exp. Psychol. A **57**, 499–521. (10.1080/02724980343000369) 15204138

[38] Rouse AA, Patel AD, Wainapel S, Kao MH. 2023 Sex differences in vocal learning ability in songbirds are linked with differences in flexible rhythm pattern perception. Anim. Behav. **203**, 193–206. (10.1016/j.anbehav.2023.05.001) 37842009 PMC10569135

[39] Burchardt LS, Knörnschild M. 2020 Comparison of methods for rhythm analysis of complex animals’ acoustic signals. PLoS Comput. Biol. **16**, e1007755. (10.1371/journal.pcbi.1007755) 32267836 PMC7141653

[40] Jedlikowski J, Brambilla M. 2017 Effect of individual incubation effort on home range size in two rallid species (Aves: Rallidae). J. Ornithol. **158**, 327–332. (10.1007/s10336-016-1373-z)

[41] Hardin JW, Hilbe JM. 2002 Generalized estimating equations. Boca Raton, FL: Chapman and Hall/CRC.

[42] Crasta JE, Thaut MH, Anderson CW, Davies PL, Gavin WJ. 2018 Auditory priming improves neural synchronization in auditory-motor entrainment. Neuropsychologia **117**, 102–112. (10.1016/j.neuropsychologia.2018.05.017) 29792887

[43] Henry CS, Wells MLM. 2006 Testing the ability of males and females to respond to altered songs in the dueting green lacewing, Chrysoperla plorabunda (Neuroptera: Chrysopidae). Behav. Ecol. Sociobiol. **61**, 39–51. (10.1007/s00265-006-0235-8)

[44] Henry MJ, Cook PF, de Reus K, Nityananda V, Rouse AA, Kotz SA. 2021 An ecological approach to measuring synchronization abilities across the animal kingdom. Phil. Trans. R. Soc. B **376**, 20200336. (10.1098/rstb.2020.0336) 34420382 PMC8380968

[45] Barmatz H, Klein D, Vortman Y, Toledo S, Lavner Y. 2018 Segmentation and analysis of bird trill vocalizations. In Int. Conf. on the Science of Electrical Engineering in Israel (ICSEE), pp. 1–5. Eilat, Israel: Institute of Electrical and Electronics Engineers.

[46] Méndez-Cárdenas MG, Zimmermann E. 2009 Duetting—a mechanism to strengthen pair bonds in a dispersed pair‐living primate (Lepilemur edwardsi)? Am. J. Phys. Anthropol. **139**, 523–532. (10.1002/ajpa.21017) 19280671

[47] Tobias JA, Sheard C, Seddon N, Meade A, Cotton AJ, Nakagawa S. 2016 Territoriality, social bonds, and the evolution of communal signaling in birds. Front. Ecol. Evol. **4**. (10.3389/fevo.2016.00074)

[48] Adret P. 2022 Developmental plasticity in primate coordinated song: parallels and divergences with duetting songbirds. Front. Ecol. Evol. **10**. (10.3389/fevo.2022.862196)

[49] Buck J. 1988 Synchronous rhythmic flashing of fireflies. II. Q. Rev. Biol. **63**, 265–289. (10.1086/415929) 3059390

[50] Kuspiel M, Kingma SA, Vermeulen H, Naguib M. 2024 Pair-coordinated calling: Eurasian magpies respond differently to simulated intruder pairs that overlap or alternate their calls. Ethology **130**, e13515. (10.1111/eth.13515)

[51] Podos J. 1997 A performance constraint on the evolution of trilled vocalizations in a songbird family (Passeriformes: Emberizidae). Evolution **51**, 537–551. (10.1111/j.1558-5646.1997.tb02441.x) 28565357

[52] Takahashi DY, Narayanan DZ, Ghazanfar AA. 2013 Coupled oscillator dynamics of vocal turn-taking in monkeys. Curr. Biol. **23**, 2162–2168. (10.1016/j.cub.2013.09.005) 24139740

[53] de Reus K, Soma M, Anichini M, Gamba M, de Heer Kloots M, Lense M, Bruno JH, Trainor L, Ravignani A. 2021 Rhythm in dyadic interactions. Phil. Trans. R. Soc. B **376**, 20200337. (10.1098/rstb.2020.0337) 34420383 PMC8380972

[54] Hartbauer M, Römer H. 2016 Rhythm generation and rhythm perception in insects: the evolution of synchronous choruses. Front. Neurosci. **10**, 223. (10.3389/fnins.2016.00223) 27303257 PMC4885851

[55] van der Vleuten BJR, Hovenkamp VA, Varkevisser JM, Spierings MJ. 2024 Context-dependent rhythmicity in chimpanzee displays. Proc. R. Soc. B **291**, 20242200. (10.1098/rspb.2024.2200) PMC11614530 39626754

[56] Ravignani A, Verga L, Greenfield MD. 2019 Interactive rhythms across species: the evolutionary biology of animal chorusing and turn‐taking. Ann. NY Acad. Sci. **1453**, 12–21. (10.1111/nyas.14230) 31515817 PMC6790674

[57] Elie JE, Hoffmann S, Dunning JL, Coleman MJ, Fortune ES, Prather JF. 2019 From perception to action: the role of auditory input in shaping vocal communication and social behaviors in birds. Brain Behav. Evol. **94**, 51–60. (10.1159/000504380) 31805560

[58] Jedlikowski J, Ręk P. 2025 Dataset from rhythmic coordination in rapid duets of little crakes: cooperative signalling without shared experience. AMUReD research data repository. (10.60629/cpjw-t504) PMC12289191 40695354

[59] Jedlikowski J, Ręk P. 2025 Supplementary material from: Rhythmic coordination in rapid duets of little crakes: cooperative signalling without shared experience. Figshare. (10.6084/m9.figshare.c.7893932) PMC12289191 40695354

